# Methenamin-3-chlorallylchlorid: MAK-Begründung, Nachtrag

**DOI:** 10.34865/mb408031kskd10_1ad

**Published:** 2025-03-31

**Authors:** Andrea Hartwig, MAK Commission

**Affiliations:** 1 Institut für Angewandte Biowissenschaften. Abteilung Lebensmittelchemie und Toxikologie. Karlsruher Institut für Technologie (KIT) Adenauerring 20a, Geb. 50.41 76131 Karlsruhe Deutschland; 2 Ständige Senatskommission zur Prüfung gesundheitsschädlicher Arbeitsstoffe. Deutsche Forschungsgemeinschaft, Kennedyallee 40, 53175 Bonn, Deutschland. Weitere Informationen: Ständige Senatskommission zur Prüfung gesundheitsschädlicher Arbeitsstoffe | DFG

**Keywords:** Methenamin-3-chlorallylchlorid, Nase, oberer Atemtrakt, Reizwirkung, Kanzerogenität, Formaldehydabspalter, Strukturanalogie, Keimzellmutagenität

## Abstract

The German Senate Commission for the Investigation of Health Hazards of Chemical Compounds in the Work Area (MAK Commission) summarized and re-evaluated the data for methenamine 3-chloroallylchloride [4080-31-3 ( *cis* /*trans*), 51229-78-8 (*cis*)] to derive an occupational exposure limit value (maximum concentration at the workplace, MAK value) considering all toxicological end points. Relevant studies were identified from a literature search and also unpublished study reports were used. Methenamine 3-chloroallylchloride releases formaldehyde in aqueous solution. The effects are therefore attributed to the hydrolysis products formaldehyde and 3-chloroprop-2-en-1-amine. There are no studies available that investigated the carcinogenicity, toxicity and genotoxic potential of methenamine 3-chloroallylchloride in the upper respiratory tract or nose, which are the likely target organs. The substance has mutagenic and clastogenic potential in vitro, presumably due to the release of formaldehyde. Formaldehyde was classified in Carcinogen Category 4 because it induces tumours in nasal tissues at concentrations that exceed their detoxification capacity. As a formaldehyde releaser, the substance could be classified in Carcinogen Category 4. However, because it is not possible to derive a MAK value for methenamine 3-chloroallylchloride, the substance has been assigned to Carcinogen Category 2 with the footnote “Prerequisite for Category 4 in principle fulfilled, but insufficient data available for the establishment of a MAK or BAT value”. As no data are available for the systemic bioavailability of methenamine 3-chloroallylchloride and the formaldehyde that is released in tissues by hydrolysis, there is no experimental evidence that the formaldehyde reaches the germ cells. Therefore, methenamine 3-chloroallylchloride has been classified in Category 3 B for germ cell mutagens. A new developmental toxicity study that was carried out according to OECD Test Guideline 414 does not confirm the foetal eye defects found by an earlier study. As no MAK value has been derived, the substance is no longer assigned to a pregnancy risk group. Current clinical findings confirm that frequent or regular contact with methenamine-3-chloroallyl chloride can lead to sensitization. Therefore, the “Sh” designation has been retained. There are no data for respiratory sensitization. Skin contact is not expected to contribute significantly to systemic toxicity.

**Table:** 

**MAK-Wert**	**–**
**Spitzenbegrenzung**	**–**

**Hautresorption**	**–**
**Sensibilisierende Wirkung (2012)**	**Sh**
**Krebserzeugende Wirkung (2023)**	**Kategorie 2^a)^**
**Fruchtschädigende Wirkung**	**–**
**Keimzellmutagene Wirkung (2023)**	**Kategorie 3 B**

**BAT-Wert**	**–**

Synonyma	1-(3-Chlorallyl)-3,5,7-triaza-1-azoniaadamantanchlorid Hexamethylentetramin-3-chlorallylchlorid *cis*-Isomer: *cis*-1-(3-Chlorallyl)-3,5,7-triaza-1-azoniaadamantanchlorid
Chemische Bezeichnung (IUPAC-Name)	*cis*-Isomer: 1-[(*Z*)-3-Chlorprop-2-enyl]-3,5,7-triaza-1-azoniatricyclo[3.3.1.13,7]decanchlorid
CAS-Nr.	4080-31-3 (*cis*/*trans*) 51229-78-8 (*cis*)
Formel	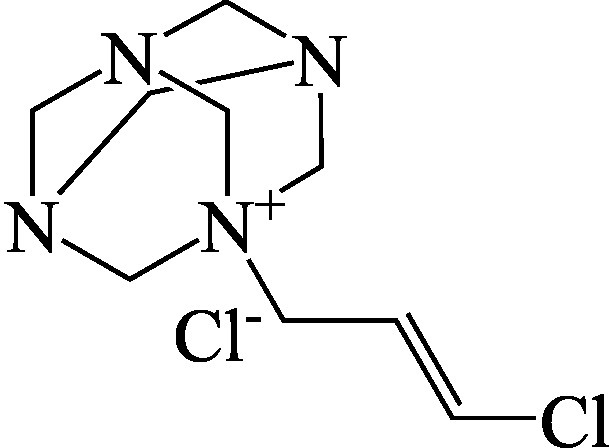
	C_9_H_16_Cl_2_N_4_
Molmasse	251 g/mol
Schmelzpunkt	151 °C (ber.; US EPA 2023)
Siedepunkt	223 °C (k. A. zum Druck, ber.; US EPA 2023)
Dampfdruck bei 25 °C	1,33 × 10^-7^ hPa (Hartwig 2012) 1,74 × 10^-6^ hPa (*cis*-Methenamin-3-chlorallylchlorid, Europäische Kommission 2019) 5,59 × 10^-7^ hPa (*cis*/*trans*-Methenamin-3-chlorallylchlorid, ECCC und Health Canada 2021)
Einsatzverbote	Kosmetika (Europäische Kommission 2019); Schutzmittel für Bearbeitungs- und Schneidflüssigkeiten; Schutzmittel für Produkte während der Lagerung; Schleimbekämpfungsmittel (Europäische Kommission 2023)

^a)^ Voraussetzung für Kategorie 4 prinzipiell erfüllt, aber Daten für MAK- oder BAT-Wert-Ableitung nicht ausreichend

Hinweis: Der Stoff ist ein Formaldehydabspalter.

Bisher liegt eine Begründung (Hartwig 2012) vor. Es erfolgt eine Neubewertung der Daten zur Formaldehydabspaltung nach aktuellem Vorgehen der Kommission (DFG 2024). Unter diesem Aspekt werden in diesem Nachtrag nur die bewertungsrelevanten Endpunkte reevaluiert.

Der Nachtrag basiert im Wesentlichen auf der Bewertung von SCCS (2011). Zitierte unveröffentlichte toxikologische Studien von Firmen wurden der Kommission zur Verfügung gestellt.

Methenamin-3-chlorallylchlorid liegt als Mischung der *cis*-/*trans*-Isomeren vor. Es wurde kein gravierender Unterschied der Toxizität beider Isomere beobachtet (Hartwig 2012), sodass in diesem Nachtrag Studien mit beiden Isomeren einbezogen werden.

## 1 Allgemeiner Wirkungscharakter

Methenamin-3-chlorallylchlorid ist ein Formaldehydabspalter. Formaldehyd ist bei inhalativer Exposition kanzerogen an der Nase von Ratten. Eine kanzerogene Wirkung an der Nase ist somit bei inhalativer Exposition gegen Methenamin-3-chlorallylchlorid zu erwarten, auch wenn keine Untersuchungen dazu vorliegen.

Methenamin-3-chlorallylchlorid ist schwach reizend an Haut und Auge.

In In-vitro-Testsystemen treten mutagene und klastogene Wirkungen auf. Schlundsondengaben an Mäusen führen nicht zu einer Erhöhung an Mikronuklei im Knochenmark.

Methenamin-3-chlorallylchlorid weist ein hautsensibilisierendes Potenzial auf.

Schlundsondengaben verursachen keine substanzbedingten entwicklungstoxischen Effekte.

Studien mit wiederholter inhalativer Exposition und zur Kanzerogenität fehlen.

## 2 Wirkungsmechanismus

In wässrigen Lösungen spaltet Methenamin-3-chlorallylchlorid Formaldehyd ab (Hartwig 2012). Es entstehen bei vollständiger Hydrolyse bis zu sechs Mol Formaldehyd pro Mol Methenamin-3-chlorallylchlorid, dazu ein Mol 3-Chlorprop-2-en-1-amin.

Die biozide Wirkung verläuft über Formaldehyd. Der Anteil der Formaldehydfreisetzung ist abhängig von der Konzentration an Methenamin-3-chlorallylchlorid in der wässrigen Lösung, dem pH-Wert, der Zeit und der Temperatur (Hartwig 2012).

Formaldehyd wirkt stark reizend nach inhalativer Exposition (Greim 2000). Von einer Formaldehydfreisetzung an den Schleimhäuten der Atemwege durch spontane Hydrolyse des Methenamin-3-chlorallylchlorids ist auszugehen.

Die sensibilisierende Wirkung von Methenamin-3-chlorallylchlorid ist auf Formaldehyd zurückzuführen (Hartwig 2012).

Einige Monohalogenalkene bilden DNA-Addukte nach Cytochrom-P450-vermittelter Bio*trans*formation zu Epoxiden. Eine Modellierung (OECD QSAR Toolbox) ergab eine möglich Bildung von Epoxidmetaboliten aus *cis*/*trans*-Methenamin-3-chlorallylchlorid unter In-vitro-, jedoch nicht unter In-vivo-Bedingungen (ECCC und Health Canada 2021).

## 3 Toxikokinetik und Metabolismus

Die Hydrolyse eines Formaldehydabspalters verstärkt sich durch die zunehmende Verdünnung im wässrigen Milieu des Atemtraktes in Abhängigkeit von der stoffspezifischen Hydrolysegeschwindigkeit. Die Formaldehydfreisetzung von *cis*-Methenamin-3-chlorallylchlorid wurde bei verschiedenen pH-Werten und Temperaturen bestimmt. Eingesetzt wurden je 10 mg Methenamin-3-chlorallylchlorid in 10 ml 0,1 M Phosphatpuffer. Die Formaldehydfreisetzung verlief bei den pH-Werten 4, 7 und 10 mit ähnlichen Halbwertszeiten. Bei 37 °C wurde etwa 25 % mehr Formaldehyd freigesetzt als bei 25 °C (Lv et al. 2015). Eine Halbwertszeit wurde nicht angeben, sie wurde aus einer Abbildung abgeschätzt. Auf Nachfrage teilten die Autoren mit, dass die fehlende Einheit der Y-Achse mg/l wäre. Somit wurden nach 24 Stunden (1440 Minuten) ca. 25 mg Formaldehyd/l freigesetzt bei einer Anfangskonzentration von 1000 mg Methenamin-3-chlorallylchlorid/l (Cheng 2023). Damit liegt die Halbwertszeit bei mehr als 24 Stunden. Die Studie weist Mängel in der Durchführung der Formaldehydbestimmung und der Darstellung auf.

Für **Diazolidinharnstoff** und **Dimethyloldimethyl (DMDM)-hydantoin, **deren Formaldehydfreisetzung von Lv et al. (2015) ebenfalls bestimmt wurde, wurden in anderen Veröffentlichungen deutlich kürzere Halbwertszeiten von 12 Stunden (20 °C) bzw. 10,7 Stunden (35 °C) bei pH 7 berichtet (ECHA 2020, 2023 a). Auffällig ist zudem, dass Lv et al. (2015) bei pH 9 im Vergleich zu pH 7 keine Steigerung der Formaldehydfreisetzung des DMD-Hydantoins angeben, während laut REACH-Registrierungsdossier die Halbwertszeit mit < 1 Stunde deutlich kürzer ist. Weiterhin ist für **Hexamin** (Hexamethylentetramin, Methenamin) im sauren Bereich die Halbwertszeit deutlich kürzer (ECHA 2022) als die von Lv et al. (2015) gemessene. Diese Unterschiede und der nicht zu erwartende Verlauf der Formaldehydfreisetzung in Form einer „Sättigungskurve“, deuten auf eine unzureichende Formaldehydbestimmung bei Lv et al. (2015) hin. In der Studie von Lv et al. (2015) ist durchgängig auffällig, dass die untersuchten Substanzen kaum eine pH-abhängige Formaldehydfreisetzung zeigen, mit Ausnahme von Paraformaldehyd.

Analysen (Polarografie) verdünnter wässriger Lösungen von Methenamin-3-chlorallylchlorid zeigten eine langsame Formaldehydfreisetzung über einen weiten pH-Bereich (pH 4–pH 10). Nach 24 Stunden wurden aus 500 µg Methenamin-3-chlorallylchlorid bei pH 6 133 µg und bei pH 8 99 µg Formaldehyd freigesetzt. Das entspricht 31,5 bzw. 27,6 % der Menge von 359 µg Formaldehyd, die maximal freigesetzt werden könnte. Die Formaldehydfreisetzung aus 500 µg Methenamin-3-chlorallylchlorid betrug nach sieben Tagen bei pH 6 165 µg (46,0 %) und bei pH 8 131 µg (36,5 %) (Scott und Wolf 1962). Die Analytik entspricht nicht heutigen Standards. Eine Verflüchtigung von Formaldehyd während der Probenentnahme kann nicht ausgeschlossen werden.

**Fazit**: Die Studien von Lv et al. (2015) und Scott und Wolf (1962) werden als nicht valide bewertet. Verlässliche Hydrolysedaten liegen nicht vor. Zur Formaldehydfreisetzung in der Lunge liegen ebenfalls keine Daten vor.

### 3.1 Aufnahme, Verteilung, Ausscheidung

Nach einmaliger oraler Gabe von 5 oder 75 mg Methenamin-3-chlorallylchlorid/kg KG resorbierten Ratten 84–88 %. Zur inhalativen Aufnahme gibt es keine Untersuchungen (Hartwig 2012).

In-vivo- oder In-vitro-Daten zur transdermalen Aufnahme beim Menschen liegen für Methenamin-3-chlorallylchlorid nicht vor. Die mit Modellen für eine 2%ige nicht mehr reizende wässrige Lösung berechnete maximale Aufnahme über die Haut unter Standardbedingungen (eine Stunde, 2000 cm^2^) betrug ca. 8 mg (Hartwig 2012).

Nach dermaler okklusiver 48-stündiger Gabe von 5 oder 75 mg ^14^C-*cis*-Methenamin-3-chlorallylchlorid (^14^C-Markierung an Seitengruppe oder Ring)/kg KG (1- bzw. 50%ige Lösung) betrug die Aufnahme bei weiblichen Fischer-Ratten 1–2 % (European Commission 2011; Hartwig 2012). Angaben zu früheren Messzeitpunkten wurden nicht gemacht.

In einer Studie nach OECD-Prüfrichtlinie 427 wurde vier F344-Ratten jeweils 5 mg ^14^C-*cis*/*trans*-Methenamin-3-chlorallylchlorid (^14^C-Markierung an Seitengruppe oder Ring)/kg KG okklusiv 48 Stunden lang dermal verabreicht. Davon wurden etwa 3 % als ^14^CO_2_ abgeatmet und 1,6 % (Seitengruppe) bzw. 2,2 % (Ring) wurden im Urin gefunden. Es werden widersprüchliche Angaben zwischen der in der Originalliteratur angegebenen Tabelle und dem Text angemerkt. Der maximal resorbierte Anteil beläuft sich auf 10 %, was vermuten lässt, dass das *cis*/*trans*-Gemisch besser aufgenommen wird, als *cis*-Methenamin-3-chlorallylchlorid (s. o.; European Commission 2011).

### 3.2 Metabolismus

In den Ausscheidungen von weiblichen F344-Ratten, denen 5 mg ^14^C-*cis*/*trans*-Methenamin-3-chlorallylchlorid-SC (Seitengruppe markiert)/kg KG dermal oder oral (Angaben unklar) appliziert wurde, konnten 13 Metaboliten detektiert werden. Drei Metaboliten (je > 5 % der verabreichten Dosis) im Urin wurden als *cis*- und *trans*-Isomere von 3-Chlorallyl-1-amin und 3,3-Dimercapturat von 1-Propanol identifiziert. In den Ausscheidungen von Ratten, denen ^14^C-*cis*/*trans*-Methenamin-3-chlorallylchlorid-RL (Ring markiert) verabreicht wurde, wurden Signale von neun Metaboliten gefunden. Mit dem Urin wurde mehr als 5 % der verabreichten Dosis in Form von zwei Metaboliten ausgeschieden, die jedoch nicht identifiziert werden konnten (Abschnitt 3.1; European Commission 2011).

Der metabolische Abbau von ^14^C-*cis*-Methenamin-3-chlorallylchlorid-RL (Ring markiert) war vom Verabreichungsweg abhängig. Die Uringehalte von *cis*- und *trans*-3-Chlorallyl-1-amin (es bleibt unklar woher das *trans*-Isomer stammen soll) lagen bei den Tieren, die Methenamin-3-chlorallylchlorid oral verabreicht bekamen, bei mehr als 5 % der verabreichten Dosis. Diese Metaboliten konnten jedoch nicht in den Urinproben der Tiere nach dermaler Applikation bestimmt werden (European Commission 2011).

## 4 Erfahrungen beim Menschen

Methenamin-3-chlorallylchlorid wirkt hautsensibilisierend (Hartwig 2012). Zu weiteren Endpunkten liegen keine Daten vor.

## 5 Tierexperimentelle Befunde und In-vitro-Untersuchungen

### 5.1 Akute Toxizität

Die Daten zur akuten Toxizität nach inhalativer und oraler Gabe sind bei Hartwig (2012) dargestellt.

#### Dermale Aufnahme

Die dermale LD_50_ betrug 605 mg Methenamin-3-chlorallylchlorid/kg KG, nachdem zwei Kaninchen pro Geschlecht eine 50%ige wässrige Lösung in Dosen von 250–2000 mg Methenamin-3-chlorallylchlorid/kg KG für 24 Stunden okklusiv appliziert wurde. Es traten Reizwirkungen bis hin zu Nekrosen auf. Nach 6,5 Stunden okklusiver Applikation einer 50%igen wässrigen Lösung starb keines der Tiere bis zur höchsten Dosis von 2000 mg/kg KG. In einer weiteren Studie wurde 252–3980 mg Methenamin-3-chlorallylchlorid/kg KG (Konzentration n. a.) je fünf weiblichen und männlichen Kaninchen auf die intakte Haut aufgetragen. Zusätzlich wurden jeweils drei Kaninchen gegen 2000 mg/kg KG und jeweils drei Kaninchen gegen pulverisiertes Methenamin-3-chlorallylchlorid in einer Dosis von 3980 mg/kg KG auf intakter oder abradierter Haut exponiert. Die kombinierte LD_50_ für die gesamte Gruppe betrug 565 mg/kg KG. Zwei weitere Studien, die nicht näher beschrieben wurden, führten zu dermalen LD_50_-Werten von 150 und 923 mg/kg KG für Kaninchen bzw. > 2000 mg/kg KG für die Ratte (Hartwig 2012).

### 5.2 Subakute, subchronische und chronische Toxizität

#### 5.2.1 Inhalative Aufnahme

Hierzu liegen keine Daten vor.

#### 5.2.2 Orale Aufnahme

Hierzu liegen keine neuen Daten vor.

#### 5.2.3 Dermale Aufnahme

Neben der bei Hartwig (2012) beschriebenen Studie mit 13-wöchiger dermaler Applikation an Kaninchen von *cis*/*trans*-Methenamin-3-chlorallylchlorid (systemische Wirkung: NOAEL 1000 mg/kg KG und Tag, Reizwirkung: NOAEL 50 mg/kg KG und Tag) wurden in einer weiteren 13-wöchigen Studie an Kaninchen mit Dosen von 1,04; 10,5 oder 31,3 mg *cis*/*trans*-Methenamin-3-chlorallylchlorid/kg KG und Tag keine systemischen oder reizenden Effekte beobachtet (European Commission 2011).

Nach 90-tägiger dermaler semiokklusiver Applikation von 0, 100, 400 oder 1200 mg *cis*/*trans*-Methenamin-3-chlorallylchlorid/kg KG und Tag an sechs Stunden pro Tag und an drei Tagen in der Woche auf geschorene und enthaarte Haut von je zehn männlichen und weiblichen Mäusen wurde keine systemische Toxizität beobachtet (European Commission 2011).

### 5.3 Wirkung auf Haut und Schleimhäute

#### 5.3.1 Haut

Bei 24-stündiger okklusiver Applikation wirkte unverdünntes Methenamin-3-chlorallylchlorid allenfalls leicht hautreizend beim Kaninchen, eine 14-tägige okklusive Behandlung mit 10%iger wässriger Lösung war leicht reizend an der intakten Haut (Hartwig 2012).

#### 5.3.2 Auge

Methenamin-3-chlorallylchlorid wirkte unverdünnt am Auge von Kaninchen allenfalls schwach reizend (Hartwig 2012). 

### 5.4 Allergene Wirkung

#### 5.4.1 Hautsensibilisierende Wirkung

Den experimentellen Untersuchungen am Tier zufolge weist Methenamin-3-chlorallylchlorid ein hautsensibilisierendes Potenzial auf (Hartwig 2012).

#### 5.4.2 Atemwegssensibilisierende Wirkung

Hierzu liegen keine Daten vor.

### 5.5 Reproduktionstoxizität

#### 5.5.1 Fertilität

Nach dermaler Applikation wurde bei Kaninchen eine verminderte Spermatogenese beobachtet, dieser Effekt konnte jedoch in einer Follow-up-Studie nicht bestätigt werden (Hartwig 2012).

In einer Studie nach OECD-Prüfrichtlinie 422 erhielten je zehn männliche und weibliche SD-Ratten 0, 75, 225 oder 750 mg *cis*/*trans*-Methenamin-3-chlorallylchlorid (*cis*/*trans*: 31,3 %/32,5 %; Reinheit 63,8 %)/kg KG und Tag dermal unter okklusiven Bedingungen sechs Stunden pro Tag appliziert. Den weiblichen Tieren wurde die Dosis beginnend vier Wochen vor der Verpaarung, während der Verpaarung, der Trächtigkeit und der Laktation verabreicht. Den erwachsenen männlichen Tieren wurde die Dosis zehn Wochen lang gegeben, beginnend vier Wochen vor der Verpaarung. Die Elterntiere wiesen dosisabhängige dermale Effekte wie Schuppung, Erytheme und Ödeme ab der niedrigen Dosis auf. In der mittleren Dosisgruppe war das Körpergewicht der weiblichen Tiere statistisch signifikant um 8,1 % erniedrigt. Es zeigten sich keine Änderungen der Reproduktionsindizes der behandelten Tiere (European Commission 2011).

#### 5.5.2 Entwicklungstoxizität

In einer Schlundsondenstudie wurden je 33 weiblichen F344-Ratten 0, 5, 25 oder 75 mg *cis*-Methenamin-3-chlorallylchlorid/kg KG und Tag (Vehikel Wasser) vom 6. bis 15. Tag der Trächtigkeit verabreicht. Die Inzidenz aller schwerwiegenden Fehlbildungen der Feten war in den Dosisgruppen 25 und 75 mg/kg KG und Tag statistisch signifikant höher als bei den Kontrollen. Die Mehrzahl der fehlgebildeten Feten wies Anomalien des Auges (Mikrophthalmie oder Anophthalmie) auf. Bei 25 bzw. 75 mg/kg KG und Tag hatten 5/145 (2 % der Feten, 17 % der Würfe) und 6/137 Feten (2 % der Feten und 19 % der Würfe) Mikrophthalmie. Obwohl nicht statistisch signifikant erhöht, zeigten zwei Ratten in zwei Würfen in der Gruppe der niedrigen Dosis schwerere Fehlbildungen im Vergleich zu einer Ratte in einem Wurf der Kontrollgruppe. In der Gruppe mit 5 mg/kg KG und Tag wies ein Fetus Mikrognathie und Anophthalmie auf, ein weiterer Fetus Polydaktylie und ein dritter Fetus, der im Uterus starb, eine Exenzephalie. Die Autoren der Studie leiten einen NOEL von 5 mg/kg KG ab (European Commission 2011; Hartwig 2012).

In einer anschließenden Studie sollte untersucht werden, ob die in der ersten Studie beobachteten ophthalmischen Fehlbildungen auf einen genetischen Clustereffekt oder auf *cis*-Methenamin-3-chlorallylchlorid zurückzuführen sind. Es wurden 25 oder 75 mg *cis*-Methenamin-3-chlorallylchlorid/kg KG und Tag per Schlundsonde an Ratten verabreicht. Die Muttertiere zeigten eine ähnliche Abnahme des Körpergewichts, der Körpergewichtszunahme und der Futteraufnahme wie in der ersten Studie. Bei 75 mg/kg KG und Tag wurde auch ein vermindertes mittleres fetales Körpergewicht festgestellt. Es wiesen 2/251 (0,8 % der Feten, 6,3 % der Würfe) und 2/209 Feten (1,0 % der Feten, 6,4 % der Würfe) in den Gruppen mit 25 bzw. 75 mg/kg KG und Tag Mikrophthalmie und/oder Anophthalmie auf. Die Autoren kommen zu dem Schluss, dass die Häufigkeit von Mikrophthalmie und/oder Anophthalmie ähnlich hoch wie in der historischen Kontrolle bei F344-Ratten ist und deutlich unter der Häufigkeit von Augenfehlbildungen in der ersten Studie liegt. Darüber hinaus legt die bekannte Neigung von F344-Ratten zu fetalen Augendefekten laut der Autoren nahe, dass die ursprünglichen Studienergebnisse wahrscheinlich auf einen spontan auftretenden genetischen Clustereffekt zurückzuführen sind und nicht auf Methenamin-3-chlorallylchlorid. Außerdem betonen die Autoren, dass es keine Dosis-Wirkungs-Beziehung für Mikrophthalmie und/oder Anophthalmie gab (k. w. A.; European Commission 2011).

Nach dermaler okklusiver Applikation von *cis*-Methenamin-3-chlorallylchlorid vom 6. bis 15. Trächtigkeitstag an F344-Ratten traten bei 250, jedoch nicht bei 500 mg/kg KG und Tag, mehr Resorptionen auf als bei den Kontrolltieren. Weitere reproduktionstoxische Effekte wurden nicht beobachtet (Hartwig 2012).

In der nach OECD-Prüfrichtlinie 422 durchgeführten Studie an SD-Ratten (siehe Abschnitt 5.5.1) erhielten die F1-Nachkommen nach dem Absetzen eine Woche lang dieselben dermalen Dosierungen. Es zeigte sich in der mittleren Dosisgruppe eine Abnahme der mittleren Körpergewichte der männlichen und weiblichen F1-Nachkommen während der Laktation (um 7,5–14,7 %). Das mittlere Körpergewicht der weiblichen F1-Nachkommen war am 21. Postnataltag statistisch signifikant erniedrigt im Vergleich zur Kontrolle. Aufgrund der starken Hautläsionen wurden die Tiere der höchsten Applikationsgruppe nach 17 Tagen getötet. Es zeigten sich keine Änderungen der Reproduktionsindizes der behandelten Tiere (European Commission 2011).

### 5.6 Genotoxizität

#### 5.6.1 In vitro

In-vitro-Genotoxizitätstests (bakterielle Mutagenitätstests, ein HPRT-Test an CHO-Zellen) weisen auf ein schwaches mutagenes Potenzial von Methenamin-3-chlorallylchlorid hin. In drei UDS-Tests an Rattenhepatozyten sowie in einem In-vitro/in-vivo-UDS-Test an Ratten wurde keine Induktion der DNA-Reparatur beobachtet (Hartwig 2012).

In einem Mutagenitätstest mit den Salmonella-Stämmen TA98, TA100, TA1535 und TA1537 wurde bei *cis*-Methenamin-3-chlorallylchlorid in Konzentrationen von 10, 20, 39, 78, 156 oder 313 µg/Platte ohne Zugabe eines metabolischen Aktivierungssystems und von 39, 78, 156, 313, 625 oder 1250 µg/Platte mit metabolischem Aktivierungssystem eine konzentrationsabhängige Zunahme (mindestens zweifach) an Mutationen in den Salmonella-Stämmen TA98 und TA100 beobachtet. Der Test wurde mit einer Präinkubationszeit von 20 Minuten ausgeführt. Zytotoxizität trat ohne metabolische Aktivierung bei 313 µg/Platte und mit Aktivierung bei 1250 µg/Platte auf (European Commission 2011). Die Originalstudie liegt nicht vor. Es gibt keine Angabe dazu, ab welcher Konzentration die Mutantenzahl verdoppelt war.

Mutationen wurden auch in E. coli WP2 uvrA bei Konzentrationen von 156, 313, 625, 1250 oder 5000 µg *cis*-Methenamin-3-chlorallylchlorid/Platte ohne und mit Zugabe eines metabolischen Aktivierungssystems nachgewiesen. Der Test wurde mit einer Präinkubationszeit von 20 Minuten ausgeführt. Zytotoxizität trat ohne metabolische Aktivierung bei > 2500 µg/Platte und mit Aktivierung bei 5000 µg/Platte auf (European Commission 2011). Die Originalstudie liegt nicht vor. Auch in dieser Studie fehlt die Angabe, ab welcher Konzentration eine verdoppelte Mutantenzahl auftrat.

Im Chromosomenaberrationstest mit CHL-Zellen wurde *cis*-Methenamin-3-chlorallylchlorid (Reinheit 97,5 %) in Konzentrationen von 10; 12,5; 15; 17,5 oder 20 µg/ml mit einer Inkubationsdauer von 24 und 48 Stunden sowie von 9,5; 19; 38 oder 76 µg/ml mit einer Inkubationsdauer von sechs Stunden eingesetzt. Ein konzentrationsabhängiger Anstieg an Chromosomenaberrationen wurde ohne Zugabe eines metabolischen Aktivierungssystems nach 24-stündiger und mit Zugabe nach sechsstündiger Inkubation beobachtet. Unter allen Behandlungsbedingungen, aber überwiegend nach 24- und 48-stündiger Behandlungsdauer ohne metabolisches Aktivierungssystem, war die Anzahl an polyploiden Zellen erhöht. Eine 50%ige Wachstumshemmung trat bei geschätzten 12 µg/ml ohne metabolische Aktivierung nach 48-stündiger Inkubation und bei 38 µg/ml mit metabolischer Aktivierung nach sechsstündiger Inkubation auf (k. w. A.; European Commission 2011). Damit zeigt Methenamin-3-chlorallylchlorid in vitro eine klastogene Wirkung. Die Originalstudie liegt nicht vor. Es fehlt die Angabe, ab welchen Konzentrationen die Induktion der Chromosomenaberrationen statistisch signifikant erhöht war.

Primäre Lymphozytenkulturen männlicher Sprague-Dawley-Ratten wurden vier Stunden gegen *cis*-Methenamin-3-chorallylchlorid (Reinheit 98,1 % ± 0,8 %) exponiert. Es wurden zwei unabhängige Tests durchgeführt. Die Auswertung erfolgte nach 24 und nur im zweiten Test auch nach 48 Stunden. Ohne Zugabe eines metabolischen Aktivierungssystems wurden Konzentrationen von 5; 16,7 und 50 µg/ml ausgewertet. Mit Zugabe des metabolischen Aktivierungssystems wurden im ersten Test 1,67 und 5 µg/ml, im zweiten 0,5; 1,67 und 5 µg/ml (Untersuchung der hohen Konzentration erfolgte auch nach 48 Stunden) eingesetzt und analysiert. Die jeweils höchste Konzentration führte zu einer 50%igen Abnahme des Mitoseindex. In beiden Tests wurde kein statistisch signifikanter Anstieg an Zellen mit Chromosomenaberrationen beobachtet, auch polyploide Zellen waren nicht erhöht (k. w. A.; European Commission 2011). Die Originalstudie liegt nicht vor. Damit zeigte Methenamin-3-chlorallylchlorid in diesem Test mit primären Lymphozytenkulturen keine klastogene Wirkung. Die Anzahl der ausgezählten Zellen ist jedoch nicht angegeben. 

Der in der Tabelle 1 dargestellte Vergleich von Methenamin-3-chlorallylchlorid und Formaldehyd zeigt eine übereinstimmende mutagene Wirkung in den bakteriellen Mutagenitätstests mit den Salmonella-typhimurium-Stämmen TA97 und TA100, ebenfalls übereinstimmend trat keine mutagene Wirkung mit den Stämmen TA1535 und TA1537 auf. Ohne metabolische Aktivierung wurden bei Formaldehyd mit TK6-Zellen und nach metabolischer Aktivierung bei Methenamin-3-chlorallylchlorid mit CHO-Zellen Genmutationen am hprt-Lokus beobachtet. Beide Substanzen verursachten Chromosomenaberrationen. Hier besteht jedoch keine Übereinstimmung bei Lymphozyten.

**Tab. 1:** Vergleich der genotoxischen Wirkung von Formaldehyd und Methenamin-3-chlorallylchlorid

	Formaldehyd				Methenamin-3-chlorallylchlorid			
Endpunkt (Testmethode, Zellen)	wirksame Konzentration	Ergebnis		Literatur	wirksame Konzentration	Ergebnis		Literatur
		–m. A.	+m. A.			–m. A.	+m. A.	
SMT E. coli	gpt-Gen: 4 mM (ca. 1,2 µg/ml)	+/–	n. u.	Crosby et al. 1988	WP2 uvrA: k. A.	+	+	European Commission 2011
SMT (TA97, TA98)	0,3 µmol (ca. 0,01 µg/ml)	+/–	n. u.	Marnett et al. 1985	TA97: 333 µg/Platte	+/–	+/–	Hartwig 2012
SMT (TA98)	–	–	–	ECHA 2023 b	TA98: 256 µg/Platte	+/–	–	
					TA98: k. A.	+	+	European Commission 2011
SMT (TA100)	k. A.	–	n. u.	Marnett et al. 1985	333 µg/Platte	–	+/–	Hartwig 2012
SMT (TA100)	–m. A.: 200 µg/Platte +m. A.: 75 µg/Patte	+	+	ECHA 2023 b	k. A.	+	+	European Commission 2011
SMT (TA1535, TA1537)	–	–	–		–	–	–	Zeiger et al. 1988
CA (CHO)	4,9 µg/ml	+	+	Greim 2000	k. A. (CHL) Zellen	+	+	European Commission 2011
	11 µg/ml	–	+	Galloway et al. 1985				
	16 µg/ml	+/–	+					
CA (humane Lymphozyten)	7,5 µg/ml	+	+	ECHA 2023 b				
CA (Lymphozyten Ratte)					k. A. (0,5–50 µg/ml)	– bis 50 µg/ml	– bis 5 µg/ml	European Commission 2011
Genmutation HPRT (TK6)	8 × 150 µM	+	n. u.	Crosby et al. 1988				
Genmutation HPRT (CHO)		–	n. u.		125 µg/ml	–	+	Hartwig 2012
Genmutation HPRT (V79)		–	n. u.	ECHA 2023 b				

–: negatives Ergebnis; +: positives Ergebnis; +/–: Ergebnis nicht eindeutig; CA: Chromosomenaberrationstest; k. A.: keine Angabe; n. u.: nicht untersucht; SMT: Salmonella-Mutagenitätstest

**Fazit**: Da Methenamin-3-chlorallylchlorid und Formaldehyd eine ähnliche Genotoxizität zeigen, ist zu vermuten, dass durch Hydrolyse in der jeweiligen Pufferlösung des Testsystems Formaldehyd in dem Maße freigesetzt wird, dass es die Genotoxizität bewirken kann.

#### 5.6.2 In vivo

In einem Mikronukleustest am Knochenmark der CD-1-Maus wirkte *cis*/*trans*-Methenamin-3-chlorallylchlorid nach fünftägiger Schlundsondengabe von 0, 100, 333 oder 1000 mg/kg KG und Tag nicht genotoxisch (Hartwig 2012).

In einem Mikronukleustest nach OECD-Prüfrichtlinie 474 induzierte *cis*-Methenamin-3-chlorallylchlorid keine Mikronuklei in Erythrozyten aus dem Knochenmark von CD-1-Mäusen nach zweimaliger Schlundsondengabe von 0, 250, 500 oder 1000 mg/kg KG und Tag (European Commission 2011).

### 5.7 Kanzerogenität

Hierzu liegen keine Daten vor.

## 6 Bewertung

Kritische Effekte sind die kanzerogene und lokal reizende Wirkung an der Nase durch das Hydrolyseprodukt Formaldehyd und die hautsensibilisierende Wirkung bei Mensch und Tier (Hartwig 2012).

**MAK-Wert. **Da verlässliche Daten zur Hydrolyse fehlen, wird vom Worst Case, einer vollständigen Formaldehydfreisetzung beim Auftreffen im Atemtrakt, ausgegangen. Es liegen keine Humandaten oder Inhalationsstudien am Tier vor, aus denen ein MAK-Wert abgeleitet werden kann.

Der Dampfdruck von Methenamin-3-chlorallylchlorid liegt bei etwa 1 × 10^–7^ hPa (Hartwig 2012). Substanzen mit einem Dampfdruck von weniger als 10^–5^ hPa liegen in der Luft weitgehend als Aerosol vor (DFG 2024). 

Das Auftreffen von Aerosoltröpfchen kann zu einer höheren (punktuellen) lokalen Konzentration an den Schleimhäuten der Atemwege führen (Aerosol-Impaktierung). Der Formaldehydabspalter kann sich im Atemtrakt dadurch reizender als das dampfförmige Formaldehyd selbst zeigen. Am Beispiel des N,N′,N′′-Tris(β-hydroxyethyl)hexahydro-1,3,5-triazins lässt sich dieser Sachverhalt darstellen. Seine stärkere lokale Wirkung in der Lunge nach Exposition gegen 3 mg/m^3 ^als die der Hydrolyseprodukte Formaldehyd und 2-Aminoethanol ist vermutlich das Resultat einer Aerosol-Impaktierung. Der berechnete Dampfdruck des N,N′,N′′-Tris(β-hydroxyethyl)hexahydro-1,3,5-triazins beträgt 5 × 10^–8^ hPa. Formaldehyd oder 2-Aminoethanol selbst lägen theoretisch beide bei der in dieser Inhalationsstudie erhaltenen LOAEC des N,N′,N′′-Tris(β-hydroxyethyl)hexahydro-1,3,5-triazins (3 mg/m^3^) dampfförmig vor. Die unerwartet starken Effekte können hier also über eine lokal verstärkte Formaldehyd- und 2-Aminoethanol-Konzentration durch Hydrolyse nach Auftreffen der Aerosole im wässrigen Milieu des Atemtraktes erklärt werden (DFG 2024; Hartwig und MAK Commission 2023).

Entsprechend kann für Methenamin-3-chlorallylchlorid kein MAK-Wert festgesetzt werden. Eine Spitzenbegrenzung entfällt.

Bei Anwendung in verdünnten wässrigen Lösungen sollte mit einer hydrolytischen Spaltung zu Formaldehyd gerechnet und daher der MAK-Wert für Formaldehyd (Greim 2000; Hartwig 2010) eingehalten werden.

**Krebserzeugende Wirkung. **Es liegen keine Untersuchungen der krebserzeugenden Wirkung für Methenamin-3-chlorallylchlorid vor. Die Tests zur genotoxischen Wirkung in vitro zeigen eine mutagene Wirkung in bakteriellen Tests und klastogene Effekte im Chromosomenaberrationstest. In-vivo-Mikronukleustests mit oraler Gabe an der Maus verlaufen jedoch negativ. Die Genotoxizität am wahrscheinlichen Zielorgan Nase aufgrund der Formaldehydfreisetzung kann nicht beurteilt werden, da Inhalationsstudien fehlen.

Die lokale Kanzerogenität von Formaldehyd ist ausführlich dokumentiert (Greim 2000). Formaldehyd ist in Kanzerogenitäts-Kategorie 4 eingestuft, da es bei Konzentrationen, die die Entgiftungskapazitäten des Nasengewebes überschreiten, dort kanzerogen wirkt.

Aufgrund der hydrolytischen Freisetzung von Formaldehyd ist dessen kanzerogene Wirkung im Nasengewebe auch bei inhalativer Exposition gegen Methenamin-3-chlorallylchlorid zu erwarten. Die Geschwindigkeit der lokalen Freisetzung im Atemtrakt ist unbekannt, daher ist eine Formaldehyd-Wirkung in der Lunge nicht auszuschließen, wenn der Abbau langsamer ist als die Anflutung.

Aufgrund der lokalen kanzerogenen Wirkung von Formaldehyd könnte Methenamin-3-chlorallylchlorid in Analogie zu Formaldehyd in Kanzerogenitäts-Kategorie 4 eingestuft werden. Da jedoch kein MAK-Wert für Methenamin-3-chlorallylchlorid abgeleitet werden kann, wird der Stoff der Kanzerogenitäts-Kategorie 2 zugeordnet und erhält die Fußnote „Voraussetzung für Kategorie 4 prinzipiell erfüllt, aber Daten für MAK- oder BAT-Wert-Ableitung nicht ausreichend“.

**Fruchtschädigende Wirkung. **Da kein MAK-Wert abgeleitet wird, entfällt die Zuordnung zu einer Schwangerschaftsgruppe. In einer Entwicklungstoxizitätsstudie an F344-Ratten mit Schlundsondengabe von 0, 5, 25 oder 75 mg *cis*-Methenamin-3-chlorallylchlorid/kg KG und Tag vom 6. bis zum 15. Gestationstag traten ab 25 mg/kg KG und Tag Fehlbildungen der Augen auf (Abschnitt 5.5.2; Hartwig 2012). In einer nachfolgenden Entwicklungstoxizitätsstudie nach OECD-Prüfrichtlinie 414 am selben Rattenstamm lagen die Inzidenzen an Augenfehlbildungen im Bereich der historischen Kontrollen und deutlich unter den Inzidenzen in der ersten Studie. Die Autoren schlussfolgerten, dass die Ergebnisse der ersten Studie wahrscheinlich auf einen spontan auftretenden genetischen Clustereffekt zurückzuführen sind und nicht auf eine spezifische Folge der Methenamin-3-chlorallylchlorid-Exposition (European Commission 2011). Die Studien sind im Original nicht zugänglich. Ein Verdacht auf eine entwicklungstoxische Wirkung (Gruppe B (Verdacht)) lässt sich daher nicht begründen.

**Keimzellmutagene Wirkung. **Methenamin-3-chlorallylchlorid wirkt an Salmonella typhimurium und E. coli mit und ohne metabolische Aktivierung sowie in einem HPRT-Test an CHO-Zellen mutagen. Im Chromosomenaberrationstest mit CHL-Zellen ließ sich eine klastogene Wirkung feststellen. Schlundsondengaben an Mäusen führen nicht zu einer Erhöhung an Mikronuklei im Knochenmark. Studien an Keimzellen fehlen.

Das Hydrolyseprodukt Formaldehyd ist in Kategorie 5 für Keimzellmutagene eingestuft. Dies bedeutet, dass durch Inhalation aufgenommenes Formaldehyd unter Einhaltung des MAK-Wertes von 0,3 ml/m^3^ nur einen sehr geringen Beitrag zum genetischen Risiko für den Menschen erwarten lässt (Greim 2000).

Theoretisch könnte Methenamin-3-chlorallylchlorid in Analogie zu Formaldehyd in die Kategorie 5 für Keimzellmutagene eingestuft werden, jedoch kann für Methenamin-3-chlorallylchlorid kein MAK-Wert aufgestellt werden.

Da Daten zur systemischen Bioverfügbarkeit des Methenamin-3-chlorallylchlorids und dem durch Hydrolyse freigesetzten Formaldehyd fehlen, liegt kein experimenteller Beleg vor, dass das freigesetzte Formaldehyd in aktiver Form die Keimzellen erreicht. Daher wird Methenamin-3-chlorallylchlorid in Kategorie 3 B für Keimzellmutagene eingestuft.

**Hautresorption. **Der Stoff war bisher nicht mit „H“ markiert. Neue Daten dazu gibt es nicht.

Auf der Basis des NOAEL nach 90-Tage-Gabe per Futter an Ratten von 4 mg/kg KG und Tag (Hartwig 2012) lässt sich eine systemisch tolerierbare Dosis am Tag für den Menschen von 43 mg wie folgt berechnen: der dem toxikokinetischen Unterschied zwischen der Ratte und dem Menschen entsprechende speziesspezifische Korrekturwert (1:4), die orale Resorption von 88 %, das Körpergewicht von 70 kg, die tägliche Exposition der Tiere im Vergleich zur 5-tägigen Exposition pro Woche am Arbeitsplatz (7:5) sowie die Übertragung der tierexperimentellen Ergebnisse auf den Menschen (1:2). Eine Zeitextrapolation wird nicht berücksichtigt, weil der kritische Effekt der verminderten Körpergewichtszunahme nur zu einzelnen Untersuchungszeitpunkten auftrat (4 mg/kg KG × 70 kg /4 (Toxikokinetikfaktor) /2 (Tier-Mensch-Extrapolation) × 0,88 (Resorption) × 7/5). Demzufolge liegt die abgeschätzte dermale Aufnahme für eine 2%ige nicht mehr reizende Lösung unter Standardbedingungen (2000 cm^2^, 1 Stunde) von 8 mg Methenamin-3-chlorallylchlorid (Abschnitt 3.1) bei 18,6 % der für den Menschen systemisch tolerierbaren Menge (43 mg) und damit unter 25 %, sodass ein möglicher Beitrag der Hautresorption zur systemischen Toxizität als vernachlässigbar bewertet wird (Hartwig 2012).

Weiterhin zeigte eine 90-Tage-Studie mit dermaler Gabe von bis zu 1000 mg/kg KG und Tag an Kaninchen keine systemische Wirkung (Hartwig 2012). Die Aufnahme über die Haut von Ratten ist bei 48-stündiger okklusiver Applikation mit maximal 10 % angegeben (Abschnitt 3.1). Angaben zur Aufnahme nach kürzeren Messzeitpunkten liegen nicht vor, daher ist 10 % ein Worst Case, der die Aufnahme unter Standardbedingungen am Arbeitsplatz vermutlich sehr deutlich überschätzt. Insgesamt würde dieser Ansatz dafürsprechen, für Methenamin-3-chlorallylchlorid weiterhin keine „H“-Markierung zu vergeben.

In einem zweiten Ansatz kann die „H“-Markierung unter dem Blickwinkel des zusätzlichen Eintrags durch transdermal aufgenommenes Methenamin-3-chlorallylchlorid und dem physiologisch bedingten Formaldehydspiegel betrachtet werden. Unter der Annahme einer raschen Hydrolyse des Methenamin-3-chlorallylchlorids mit Bildung von Formaldehyd (bei vollständiger Hydrolyse bis zu sechs Mol Formaldehyd pro Mol Methenamin-3-chlorallylchlorid) lässt sich die Zunahme des Formaldehydspiegels im Blut nach dermaler Applikation unter Standardbedingungen abschätzen:

Aus den zuvor berechneten insgesamt 8 mg Methenamin-3-chlorallylchlorid (0,032 mmol) werden 5,76 mg Formaldehyd (0,032 mmol × 6 = 0,19 mmol) über einen Zeitraum von einer Stunde freigesetzt, pro Minute demnach etwa 96 µg bzw. 120 µg/1,25 Minuten (mittlere Halbwertszeit des Formaldehyds im Blut; Kaden et al. 2010). Die transdermale Aufnahme von Methenamin-3-chlorallylchlorid in den letzten sechs Halbwertszeitintervallen führt demnach zu einer im Blut zirkulierenden Formaldehydmenge von (120 + 60 + 30 + 15 + 7,5 + 3,75) µg = 236,25 µg bzw. etwa 0,24 mg.

Der physiologische Formaldehydspiegel im Blut des Menschen beträgt etwa 2–3 mg/l (10–15 mg in 5 Liter Blut, frei und reversibel gebunden) (Heck et al. 1985). Der zusätzliche Eintrag durch transdermal aufgenommenes Methenamin-3-chlorallylchlorid ist unbedeutend, so dass der Stoff weiterhin nicht mit „H“ markiert wird.

**Sensibilisierende Wirkung. **Methenamin-3-chlorallylchlorid weist ein hautsensibilisierendes Potenzial auf. Aktuelle klinische Befunde bestätigen, dass häufiger oder regelmäßiger Kontakt mit Methenamin-3-chlorallylchlorid, welches ein Formaldehydabspalter ist, zu einer Sensibilisierung führen kann. Methenamin-3-chlorallylchlorid wird daher weiterhin mit „Sh“ markiert. Zur atemwegssensibilisierenden Wirkung liegen keine Daten vor. Eine Markierung mit „Sa“ erfolgt daher nicht.
